# Type 2 Diabetes in Obesity: A Systems Biology Study on Serum and Adipose Tissue Proteomic Profiles

**DOI:** 10.3390/ijms24010827

**Published:** 2023-01-03

**Authors:** Gemma Arderiu, Guiomar Mendieta, Alex Gallinat, Carmen Lambert, Alberto Díez-Caballero, Carlos Ballesta, Lina Badimon

**Affiliations:** 1 Cardiovascular-Program, Institut d’Investigació Biomèdica Sant Pau (IIB Sant Pau), 08041 Barcelona, Spain; 2 Centro de Investigación Biomédica en Red de Enfermedades Cardiovasculares (CiberCV), 28029 Barcelona, Spain; 3 Centro Nacional de Investigaciones Cardiovasculares (CNIC), 28029 Madrid, Spain; 4 IPSA-Instituto de Investigación Sanitaria del Principado de Asturias, 33011 Oviedo, Spain; 5 Centro Médico Teknon, Grupo Quiron Salut, 08022 Barcelona, Spain

**Keywords:** visceral adipose tissue, subcutaneous adipose tissue, diabetes, proteomic analysis

## Abstract

Obesity is associated with metabolic disorders such as insulin resistance and type 2 diabetes mellitus (T2DM), further increasing an already heightened cardiovascular risk. Here, amongst obese class III bariatric surgery patients, we have investigated the effect of T2DM in serum and in two, same patient, adipose tissue (AT) depots through proteomic profile expression analyses. Serum and AT samples from subcutaneous (SAT) and visceral (VAT) fat were collected during bariatric surgery. Bead-based targeted multiplex assay systems were used to simultaneously detect and quantify multiple targets in serum samples (targeted proteomics) and analyze changes in adipokine serum composition. AT samples were assessed through an untargeted proteomics approach. Through a systems biology analysis of the proteomic data, information on the affected biological pathways was acquired. In obese class III individuals, the presence of T2DM induced a significantly higher systemic release of ghrelin, GLP-1, glucagon, MMP3, BAFF, chitinase 3-like 1, TNF-R1 and TNF-R2, and a lower systemic release of IL-8. SAT and VAT proteomes belonging to the same patient showed significant differences in local protein content. While the proteins upregulated in VAT were indicative of metabolic dysregulation, SAT protein upregulation suggested adequate endocrine regulation. The presence of T2DM significantly affected VAT protein composition through the upregulation of dysregulating metabolic pathways, but SAT protein composition was not significantly modified. Our results show that T2DM induces metabolic dysregulation in obese individuals with changes in systemic marker levels and impairment of proteostasis in VAT but not in SAT.

## 1. Introduction

Obesity is a common and serious health condition. With an incidence that has exponentially increased over the last few decades, obesity is recognized today as the new epidemic of the 21st century [1]. Significant advances in the understanding of the pathophysiology and paracrine pathways underlying the disease have been reported in the last few years, contributing to a better understanding of its role and impact on morbidity and mortality [2]. However, several gaps in knowledge persist regarding obesity-related disease mechanisms. While a basic understanding of the metabolic changes in relation to body weight is lacking, the effect of obesity in the absence of other co-morbidities (i.e., dyslipidemia, hyperglycemia and/or hypertension) [2] is still not fully understood.

White adipose tissue (AT), once regarded as a mere energy storage reservoir, has been identified as one of the most metabolically active tissues, functioning as an active endocrine organ [3] that secretes numerous bioactive molecules and hormones called adipokines [4]. AT function and composition are depot-specific. Metabolic disorders such as insulin resistance and type 2 diabetes mellitus (T2DM) are commonly associated with the presence of visceral adipose tissue (VAT) [5,6], usually located in the intra-abdominal cavity (including visceral and mesenteric fat), whereas subcutaneous adipose tissue (SAT) (broadly distributed right under the skin) is considered less harmful or even protective [7,8]. We have previously studied the influence of T2DM and other cardiovascular risk factors on the genomic profile of resident adipose stem cells (ASCs) in different fat depots, in both humans [9,10] and experimental models [11,12,13]. Specific differences and functions were found among ASCs depending on the location of the AT depot of origin and the presence of T2DM. Here, in this hypothesis-generating, discovery study, we have aimed at uncovering the effect of T2DM on targeted blood markers and on untargeted-SAT and VAT protein proteostasis.

## 2. Results

### 2.1. Influence of Type 2 Diabetes on Serum Proteins of Obese Individuals

Forty-nine proteins related to inflammation and diabetes were investigated. A group of nonOB and nonT2DM were included as reference (Supplementary Table S1).

**Figure 1:**
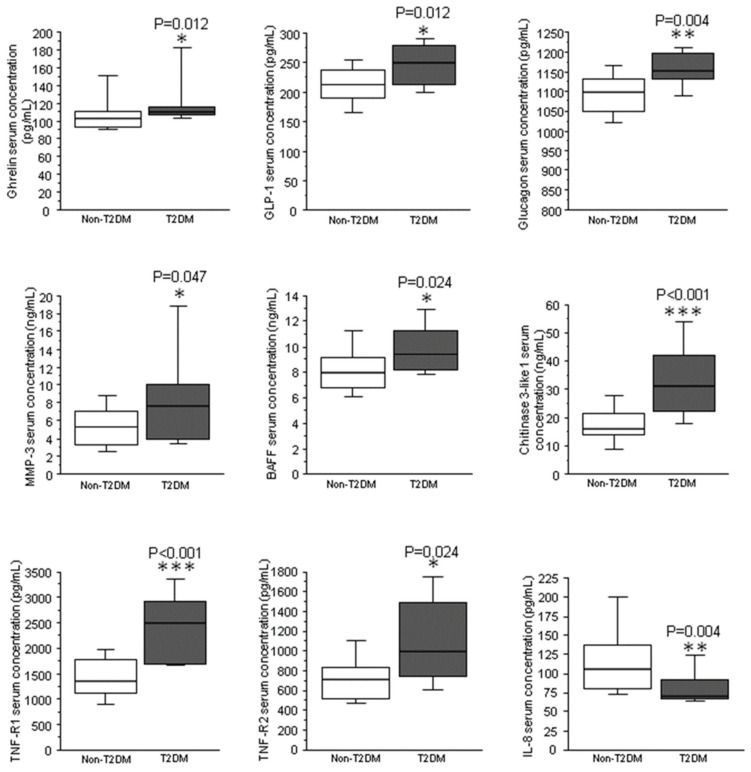
Changes in protein serum levels due to diabetes. Box-plot diagram showing the effect of type 2 diabetes on the serum proteomic profile. Data represented as mean ± SEM, statistical analysis was performed by Mann–Whitney U tests. * *p* < 0.05; ** *p* < 0.01; *** *p* < 0.001.

Compared to obese nonT2DM individuals, in obese class III (OBIII) patients, T2DM induced higher serum levels of the following 7 proteins, which are all involved in glucose metabolism, satiety and inflammation (Figure 1): ghrelin, glucagon like-peptide-1 (GLP-1), glucagon, B-cell activating factor (BAFF), chitinase 3-like, tumor necrosis factor receptor 1 (TNF-R1) and tumor necrosis factor receptor 2 (TNF-R2) and matrix metalloproteinase-3 (MMP-3). In contrast, the pro-inflammatory cytokine interleukin-8 (IL-8) showed significantly lower levels in obese T2DM versus obese nonT2DM.

**Figure 2:**
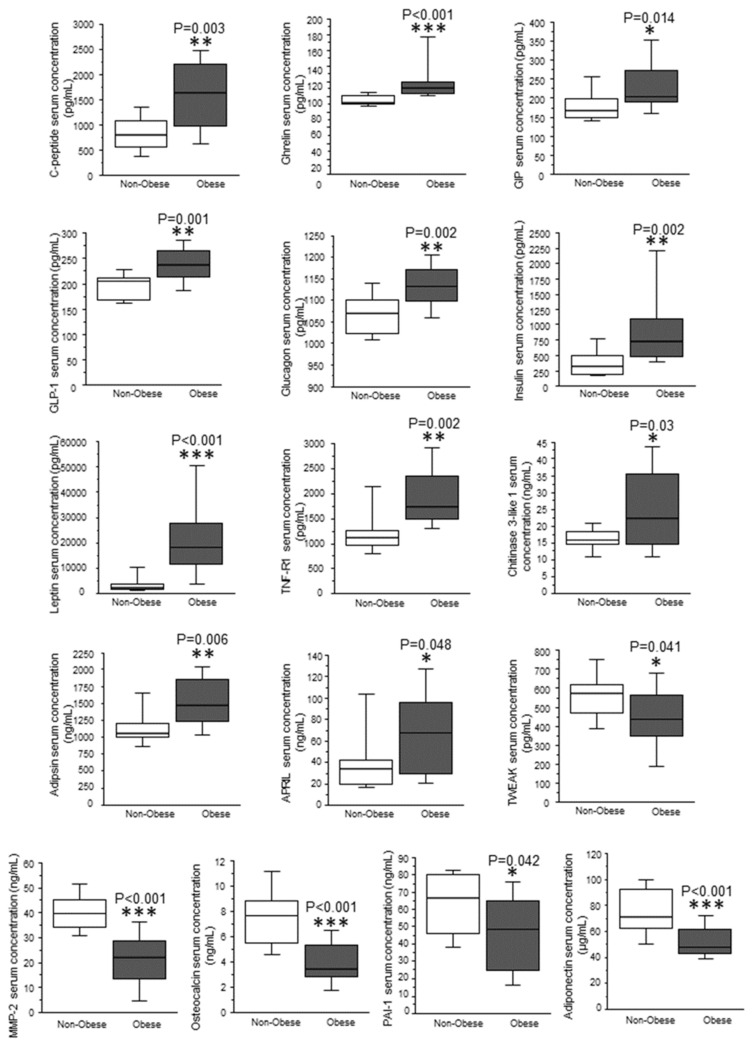
Changes in protein serum levels due to obesity. Box-plot diagram showing the effect of obesity on the serum proteomic profile. Data represented as mean ± SEM, statistical analysis was performed by Mann–Whitney U tests. * p < 0.05; ** p < 0.01; *** p < 0.001.

When comparing OBIII patients with reference control individuals (nonOB), 10 proteins appeared in significantly higher levels in the former (Figure 2): pro-inflammatory proteins such as c-peptide, chitinase 3-like 1, TNF-R1, gut-derived incretin hormones such as ghrelin, glucose-dependent insulinotropic polypeptide (GIP), GLP-1, glucagon, glucose-related adipokines such as insulin, leptin and adipsin. Three proteins were minimally affected APRIL (a proliferation-inducing ligand), PAI-1 (plasminogen activator-inhibitor-1) and TWEAK (TNF-related weak inducer of apoptosis). Compared to nonOB, three proteins were found in lower levels in OB individuals: the obesity-related adipokine adiponectin, the inflammatory proteins matrix metalloproteinase-2 (MMP-2) and osteocalcin.

Serum proteins with levels showing statistically significant differences amongst nonOB versus nonT2DM, OB versus nonT2DM and OB versus T2DM are shown in Table 1. Increased levels of proteins involved in inflammatory and metabolic pathways such as c-peptide, GLP-1, glucagon, insulin, adipsin, chitinase 3-like 1 and TNF-R1, were found in OB individuals. In contrast, OB subjects had reduced levels of adiponectin. Gut-derived incretins, ghrelin, GIP and leptin were significantly lower in the control group (nonOB and nonT2DM), whereas no changes were observed in OB-T2DM subjects. On the contrary, MMP-2 and osteocalcin serum levels were significantly higher in the nonOB and nonT2DM versus OB individuals. Additionally, osteopontin, MMP-3, TNF-R2 and BAFF levels were significantly higher in the OB-T2DM group compared to the OB-nonT2DM group, suggesting an increased inflammatory state due to T2DM. Finally, in OB-nonT2DM subjects, TWEAK levels were reduced compared to controls, and IL-8 levels were also reduced compared to both the nonOB and the OB-T2DM.

### 2.2. Differential Protein Expression between Subcutaneous and Visceral White Adipose Tissue

**Figure 3:**
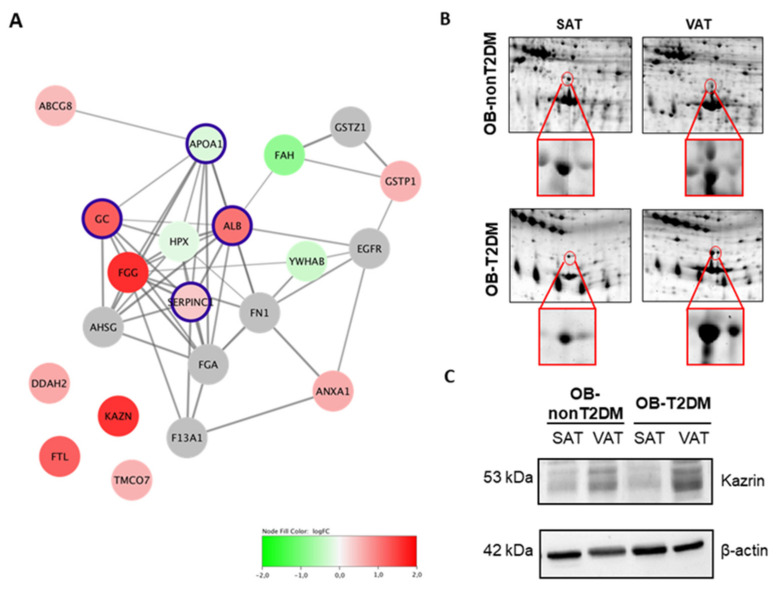
Differential proteomic signature of VAT and SAT. (**A**) Protein–protein interaction network analysis of visceral versus subcutaneous adipose tissue differentially expressed proteins. Nodes coloring represents log fold-change between VAT and SAT; gray nodes represent predicted protein interactions; nodes highlighted in purple represent predicted proteins that were detected within the proteomic analysis but did not reach significance. Red up regulated and green down regulated. (**B**) Representative two-dimensional electrophoresis image of adipose tissue with kazrin spot (MW: 86 kDa, pI: 6.6) highlighted in a circle. (**C**) Kazrin protein expression in SAT and VAT from class III obese (OB) individuals with and without type 2 diabetes (T2DM or nonT2DM). Blots are representative of 6 independent experiments.

The protein signature of white AT was resolved by 2-DE. A total of 72 non-redundant proteins were identified in the differential proteomes of SAT and VAT of OBIII patients. Since both fat depots belonged to the same donor, dietary, environmental and genetic factors can be excluded as potential sources of confounding factors (Supplementary Table S2). Comparative analyses of protein expression between SAT and VAT yielded significant differences for 10 proteins, which corresponded to different functional groups including coagulation, oxidation, energy homeostasis, chaperones and signaling pathway regulators, among others. Differences by depot considering all obese individuals showed two proteins that were down-regulated in VAT versus SAT (fumarylacetoacetase [FAH] and hemopexin [HPX]), while 10 proteins were up-regulated (ATP-binding cassette sub-family G member 8 [ABCG8], annexin A1 [ANXA1], N(G)-dimethylarginine dimethyl-aminohydrolase 2 [DDAH2], fibrinogen gamma chain [FGG], ferritin light chain [FTL], glutathione S-transferase P [GSTP1], kazrin protein (KAZN) and transmembrane and coiled-coil domain-containing protein 7 [TMCO7]) (Table 2). Differentially expressed proteins were then employed to build a PPI network to identify additional protein interactions, four of which were indeed detected in both proteomes but did not qualify for a significantly different expression (Figure 3A). Strong enrichments in Gene Ontology biological process terms associated to platelet degranulation and secretion were found for the characterized network (Table 3). Across the characterized proteomic profiles, we identified KAZN to be the most differentially expressed protein between VAT and SAT, regardless of diabetic status (Figure 3B,C).

Next, we investigated the effect of cardiovascular risk factors on the expression of the identified proteins by proteomics in the different fat depots, including the effect of glucose, triglycerides, total cholesterol, HDL-c and LDL-c. We observed that, both in VAT and SAT, total cholesterol and LDL-c levels were the parameters with the greatest impact on protein expression (Table 4).

### 2.3. Type 2 Diabetes and Obesity: Effects on the Proteome of Visceral and Subcutaneous Adipose Tissue

**Figure 4:**
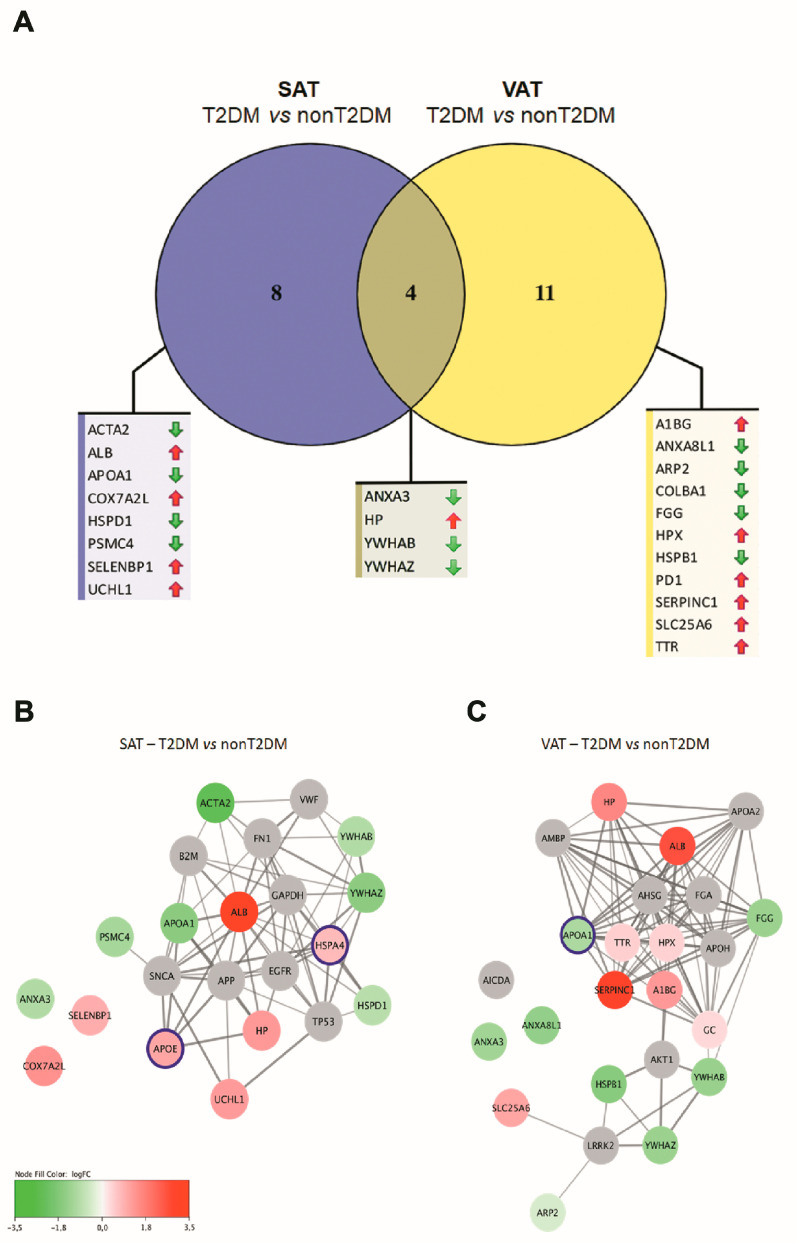
Effect of type 2 diabetes mellitus upon visceral and subcutaneous white adipose tissue proteomes. (**A**) Venn diagram of differentially regulated proteins in type 2 diabetes mellitus individuals (T2DM) compared to non-type 2 diabetes mellitus individuals (nT2DM) for VAT and SAT depots. Only proteins with significant protein expression changes (*p* < 0.05) were included, red arrows upregulated, green arrows downregulated. (**B**) Protein–protein interaction network analysis of VAT differentially expressed proteins in T2DM. (**C**) Protein–protein interaction network analysis of SAT differentially expressed proteins in T2DM. Nodes coloring represents log fold-change between T2DM and nT2DM; gray nodes represent predicted protein interactions; nodes highlighted in purple represent predicted proteins that were detected within the proteomic analysis but did not reach significance.

Changes in AT protein composition within the same type of fat depot according to T2DM presence or not were then investigated. Because the effect of T2DM on AT is different depending on its location, not all proteins with a differential profile on SAT showed differences in VAT and vice versa. T2DM was associated with significant changes in the expression of 12 proteins in SAT and 15 proteins in VAT (Table 5 and Figure 4A). Of those, four proteins were detected to be differentially expressed both in SAT and VAT: reduction in annexin A3 (ANX3), 14-3-3 protein a/b (YWHAB) and 14-3-3 protein zeta/delta (YWHAZ) and an increased level of haptoglobin (HP). Differentially expressed proteins were then employed to build PPI networks allowing for the identification of additional interacting proteins (Figure 4B,C). Noteworthy, some of the predicted additional proteins were indeed detected within the proteomic analysis but did not pass the significance threshold for differential expression. Gene Ontology biological process term enrichments were then analyzed within each network. As a result, strongly significant enrichments in secretion- and exocytosis-associated Gene Ontology terms were found for T2DM differential expression network for VAT, whereas response to stress represented the highest enrichment for SAT network (Table 6).

### 2.4. Effect of White Adipose Tissue Proteome Composition upon Serum Adipokine Signature

**Figure 5:**
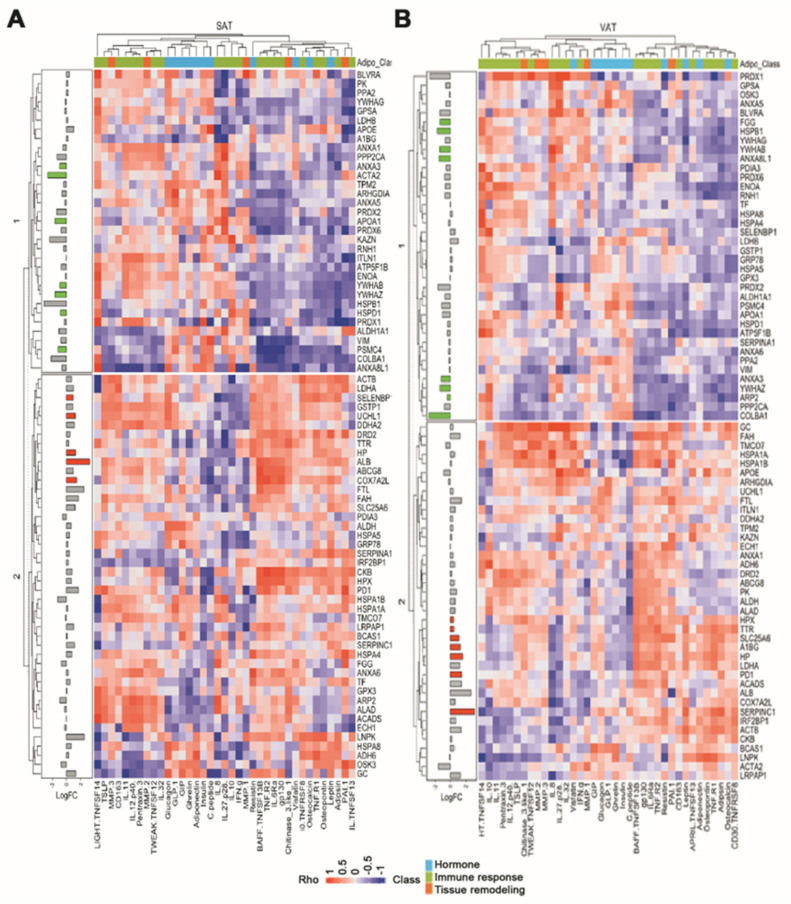
Influence of white adipose tissue proteome composition on adipokine profile. Heatmap depicting Spearman’s Rho correlation coefficient between SAT (**A**) or VAT (**B**) detected proteins (rows) and measured adipokines (columns). Log fold-change of AT between T2DM and nonT2DM individuals is represented alongside the heatmap. Colored bars indicate significance (*p* < 0.05). Rho, Spearman’s Rho correlation coefficient; LogFC, log fold-change.

Finally, we explored whether the proteomic composition of VAT and SAT affects the serum adipokine signature. With this purpose, Spearman’s Rho correlation coefficient was calculated for every pair of AT proteins/adipokines and employed to build a heatmap. Hierarchical clustering was then applied to identify regulatory modules. As a result, strong correlations were found for multiple AT proteins/adipokines pairs, and two clusters exhibiting opposite correlation coefficients were clearly identified for both VAT and SAT (Figure 5) proteomes. Remarkably, in both cases, the cluster exhibiting an overall positive correlation with the adipokines profile included all significantly up-regulated proteins in T2DM, whereas the overall negatively correlated cluster included all significantly down-regulated proteins.

## 3. Discussion

In this study, the differential effect of T2DM on same-host VAT and SAT fat depots of OBIII patients, and on their serum proteome, has been investigated for the first time through proteomics and PPI network analysis.

Obesity is an established risk factor for cardiovascular disease (CVD) [14]. However, in certain clinical scenarios, improved survival outcomes have been associated to overweight and obesity, giving rise to the “obesity paradox” [15,16]. Until now, most of the studies carried out on AT have been performed by using non-invasive imaging technologies such as magnetic resonance imaging, X-ray or ultrasound [17,18,19] as well as in different animal models. However, some limitations appear when animal findings are translated to human studies [20,21]. A limited number of human studies have compared the proteomic profile of both VAT and SAT depots of the same individual, with heterogeneous results. Pérez-Pérez et al. [22] found an increased expression of metabolism-related proteins in VAT compared with SAT, but Insener et al. did not reproduce similar findings [19]. Considering that obesity is associated with the development of insulin resistance and T2DM, but not all obese individuals become insulin resistant or diabetic, there is reason to believe serum and AT composition, or AT location, might differ in obese individuals with or without other metabolic alterations [18,23].

The sex difference in AT is an important factor to consider when studying an individuals’ risk for obesity and metabolic dysfunction [24]. There are sex differences in how AT responds to obesity leading to altered levels of lipolysis, inflammation and metabolism. Menopause has been associated with changes in AT phenotype related to metabolic dysfunction for both SAT and VAT, showing that the inflammatory profile in SAT and VAT was related to insulin resistance [25].

VAT proteomic signatures of diabetic and non-diabetic individuals have been previously compared, but only a few differences were detected [26]. However, SAT proteomic signatures in diabetic and non-diabetic individuals have not been previously investigated.

In contrast to SAT, VAT is more strongly associated to cardio-metabolic derangements [27] and accordingly, more changes in VAT compared with SAT were observed in the presence of T2DM. Likewise, changes in SAT are related to the endocrine system, whereas changes in VAT are linked to metabolic disease, supporting the involvement of VAT in metabolic disorders in comparison with that of SAT. Thus, an altered metabolic and pro-inflammatory state was observed both in AT and in serum suggesting a coordinated effect probably caused by the altered protein secretion from adipocytes to the bloodstream. Among all the identified proteins in AT, only hemopexin (HPX), actin-related protein-2 (Arp2) and 14-3-3-protein beta/alpha (YWHAB) were reduced in VAT compared with SAT. HPX and Arp2 are both only modified in the diabetic group, and both associated with adipocyte differentiation [28,29] suggesting adipocyte dysfunction in the VAT of diabetic individuals. On the other hand, a relatively increased expression of many other proteins, but perhaps most importantly KAZN and N(G)-dimethylarginine dimethylaminohydrolase 2 (DDAH2) was detected in VAT compared with SAT.

KAZN was initially identified bounded to the N-terminal domain of the cytolinker periplakin [30]. While multiple functions, ranging from regulation of desmosome assembly [31], epidermal differentiation by regulation of keratinocyte adhesion and differentiation [32] have been attributed to KAZN, its expression in AT has never been described before, and as expected, it displayed an unconnected node in the network. The described differences in KAZN protein expression across VAT and SAT suggests a novel role for this protein in VAT biology. Desmosomes are intercellular junctions of epithelia and cardiac muscle. The disruption of desmosomal adhesion is a pathological process; in fact, mutations in genes encoding desmosomal proteins have been related with arrhythmogenic right ventricular dysplasia, a heart muscle condition characterized by life-threatening arrhythmias and increased risk of sudden heart failure [33]. Likewise, the presence of KAZN in AT suggests some degree of AT dystrophy. By contrast, DDAH2, a protein narrowly related with insulin secretion [34], has been previously detected in VAT, but not in SAT; therefore, a comparison of the expression of this protein in different fat depots has not been previously published. The presence of these two proteins in both AT depots highlights the need for further investigation in adipose cell function and regulation, to further determine how fat location in obesity determines the development of further cardio-metabolic disease and the role that T2DM plays.

Besides its association with metabolic disorders, obesity seems to have an effect on oxidative stress that affects countless diseases. This might be one potential pathway linking obesity and related disorders such as T2DM and CVD [35,36]. Indeed, carbohydrate-rich diets induce oxidative stress and inflammatory states in obese individuals [35]. Additionally, Murri et al. have previously described the effect of T2DM on the visceral oxidative proteome [26]. They described an antioxidant state in the visceral fat of diabetic individuals that could act as a defense against the metabolic stress induced by insulin resistance, diabetes and hyperglycemia. In line with these, we aimed to compare the differential oxidative stress among the different fat depots, and we observed an up-regulation of different oxidation-related proteins in VAT compared with SAT, including antioxidant proteins such as peroxiredoxin-6 [37], GSTP1 [38] and the glutathione-related protein disulfide isomerase-3 (PDIA3) [39], supporting the concept that VAT from diabetic individuals could try to compensate the characteristic oxidative stress resulting from impaired glucose handling. However, not only oxidation-related proteins were up-regulated in VAT but also proteins related with coagulation, inflammation and cholesterol efflux, indicating that VAT is a much more active fat depot than SAT. Differentially expressed proteins in SAT were related to stress response, localization and motility. Differences in the biological processes detected between VAT and SAT may arise from intrinsic differences in tissue composition. Compared with SAT, VAT is more cellular, vascular, innervated and contains a larger number of inflammatory and immune cells [40]. It also exhibits a lesser preadipocyte-differentiating capacity and a greater percentage of large adipocytes. There are more glucocorticoid and androgen receptors in VAT than in SAT [41]. VAT adipocytes are more metabolically active, more sensitive to lipolysis and more insulin-resistant than SAT adipocytes. While SAT is more avid in the absorption of circulating free fatty acids and triglycerides, VAT is more sensitive to adrenergic stimulation; it has a greater capacity to generate free fatty acids and to uptake glucose [42].

In addition to the differential AT protein composition, we also observed changes at serum levels, related both to obesity and T2DM. Obesity is known to be associated with a low-grade pro-inflammatory state and many inflammatory adipokines are altered in the secretome of obese individuals compared with lean subjects [43,44]. The same is true for AT secreted proteins, where adipokines such as leptin, adiponectin and visfatin, among others, are dysregulated in response to fat accumulation [45]. Thus, as expected, the pro-inflammatory adipokines leptin, adipsin, insulin, C-peptide, chitinase 3-like 1, TNF-R1 and TNF-ligand superfamily member 13 (TNFSF13 or APRIL) were found in higher levels in the serum profile of the obese group relative to lean subjects, and osteocalcin, which improves insulin resistance by decreasing inflammation [46], was found in lower levels amongst obese individuals. Interestingly, we found that, in our cohort of obese patients, IL-8 levels were decreased in the obese T2DM patients with respect to the obese non-diabetic ones. This could be due to the management of T2DM patients in this study, as they have been prepared to undergo a bariatric surgical intervention and have been treated for diabetes. It has been described that IL-8 production and release are higher in VAT compared with SAT [47].

A narrow relation between insulin resistance, T2DM and inflammation is also generally accepted [48]. Therefore, a coordinated pro-inflammatory state is observed not only in the AT, but also in the serum proteins as a response to obesity and diabetes. However, serum protein composition is more complex than just AT secreted proteins, being also affected by the secretion of other cytokines produced by other organs such as the pancreas, the liver and the hypothalamus [49] and cells from the cardiovascular system.

At a systemic level, we have observed significantly higher levels of the gut-derived incretin hormones; specifically, obese individuals showed higher levels of ghrelin, GIP, GLP-1 and glucagon. Incretin hormones have an important role in glucose homeostasis [50]. In fact, T2DM individuals showed decreased bioavailability of these proteins. In previous studies, incretins have shown an anti-inflammatory, antioxidant and anti-apoptotic effect, and considering their role in appetite and satiety, they have also been widely associated to obesity [51]. Interestingly, serum levels of PAI-1, an inhibitor of fibrinolysis that is usually up-regulated in obesity [52], were unaltered in this study; perhaps, the hypocaloric diet prior to the actual bariatric surgery may have had an impact on their metabolic profile. However, as expected, the weight loss of those individuals (around 5 to 8% of the total body mass) was not enough to achieve a total improvement on the serum profile of OBIII subjects, and the down-regulation of adiponectin, which negatively correlates with body mass [43], and the up-regulation of adipsin and leptin, both of which are usually increased with body mass gain [45], were observed in the serum proteome of OBIII individuals.

Not only obesity, but also diabetes affects the serum proteome. However, unlike in AT composition where metabolic, oxidation, coagulation and inflammatory proteins were altered, mainly inflammatory cytokines such as IL-8, TNFSF13 B, TNFR1 and TNFR2 [40] and the metalloproteinase MMP-3 were affected in the serum of diabetic individuals, independently of the presence of obesity.

This pro-inflammatory state is, however, accompanied by the increase in insulin in T2DM individuals and adipsin levels in obese diabetic individuals. Adipsin, which is a member of the complement system, is also associated with the protection of β-cells and, therefore, positively related to insulin levels [53].

White AT is widely conceived as a metabolically active tissue with endocrine function [3]. Therefore, we hypothesized the existence of an association between the AT proteomic profile and the adipokine signature. We tested the feasibility of this idea following a correlation approach and found strong correlations for many AT protein–adipokine pairs, suggesting a functional relationship. However, as correlation does not imply causation, the existence of a high correlation coefficient between an AT protein–adipokine pair does not mean that one regulates the other, but instead, that both are regulated in the same fashion. Heatmap construction allowed us to apply hierarchical clustering to identify regulatory modules (i.e., groups of AT proteins and adipokines that are regulated following the same trend). We identified two clusters of AT proteins for both SAT and VAT. Interestingly, cluster 1 included all proteins identified to be significantly down-regulated in diabetes, whilst cluster 2 included all significantly up-regulated proteins. Furthermore, cluster 1 exhibited an overall negative correlation coefficient with nearly the half of the measured adipokines, whilst the same adipokines exhibited an overall positive correlation coefficient with the proteins included in cluster 2, for both depots. Although there is not a robust statistical approach to prove a causal relationship, this observation reinforces the existence of a link between AT and the adipokine profile, which is affected by diabetes.

## 4. Materials and Methods

### 4.1. Study Population 

This study enrolled consecutive obese class III individuals with BMI ≥ 40 scheduled for bariatric surgery (N = 38). Following standard operating procedures, prior to surgery, all individuals underwent a hypocaloric diet to reduce total body weight by 5–8%. All individuals fasted for at least 12 h overnight before entering the operating room. Written informed consent was obtained from all subjects. The study complied with the recommendations of the Declaration of Helsinki and was approved by the Centro Médico Teknon Ethics Committee.

### 4.2. Sample Collection

Blood samples were obtained during the surgical intervention in order to analyze metabolic markers and serum protein composition. Blood samples were collected in anticoagulant-free Vacutainer tubes for serum preparation. Serum fractions were separated by centrifugation at 3000× *g* at 4 °C for 20 min, aliquoted and stored at −80 °C until analyzed. Targeted serum proteomic characterization in blood was performed by the Bio-Plex Multiplex System powered by Luminex xMAP technology, a multiplex assay system to simultaneously detect and quantify multiple targets analyzed in qualified complex samples that were obtained from a group of obese non-diabetic (OB-nonT2DM) (*n* = 13) individuals and another group of obese diabetic (OB-T2DM) individuals (*n* = 14). Additionally, and for comparative purposes, plasma from control (non-obese and non-diabetic) (nonOB/nonT2DM) individuals was analyzed (*n* = 11).

**Figure 6:**
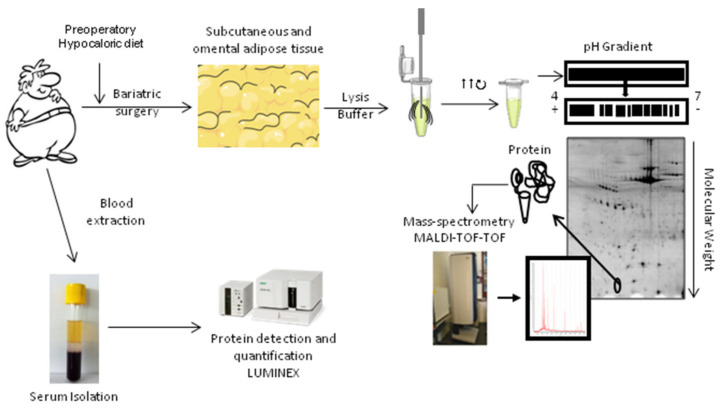
Study design. SAT and VAT from obese class III individuals (with or without T2DM) were collected. Proteins were extracted with a lysis buffer and a proteomic approach was used to identify changes in the proteomic profile due to fat location or due to the presence of T2DM.

VAT and SAT samples were also obtained from twelve class III obese patients at the time of blood extraction during surgery. These were processed for untargeted proteomics in OB-nonT2DM individuals (*n* = 6) and in OB-T2DM individuals (*n* = 6) with fully characterized demographics (Table 7 and Figure 6). AT was collected under sterile conditions; samples were washed in phosphate-buffered saline and immediately frozen in liquid N2. Samples were stored at −80 °C until protein extraction.

AT protein isolation was performed following the previously described method by Corton et al. [54] with minor modifications. In brief, AT was mechanically homogenized, diluted in lysis buffer (8.4 mol/L urea, 2.4 mol/L thiourea, 50 g/L CHAPS, 50 mmol/l DTT) and sonicated (6 cycles of 15 s). The suspension was shaken for 1 h at room temperature and centrifuged at 60,000 rpm for 1.5 h.

### 4.3. Biochemical Analysis

Blood levels of glucose, triglycerides, total cholesterol, high-density lipoprotein (HDL) and low-density lipoprotein (LDL) cholesterol were determined with a CLIMA MC-15 analyzer (RAL) (Table 1).

### 4.4. Two-Dimensional Gel Electrophoresis (2-DE)

Tissue samples were desalted using the ReadyPrep 2D Cleanup Kit (Bio Rad, Life Science, Barcelona, Spain), and proteins were diluted in urea/thiourea buffer (7 mol/L urea, 2 mol/L thiourea and 20 g/L Bio Rad, Life Science, Barcelona, Spain). The protein concentration was determined using the 2-D Quant Kit (GE Healthcare, Madrid, Spain).

Protein extracts from SAT and VAT (100 µg) were diluted in urea/thiourea buffer supplemented with 1% (*v*/*v*) IPG buffer, and 50 mmol/L DTT and then applied to IPG strips (pH 4–7; Bio-Rad) by active rehydration (12 h; 50 V). IPG strips were then equilibrated in equilibration buffer (0.1% DTT) to maintain the fully reduced state of proteins, followed by 2.5% iodoacetamide to prevent reoxidation of thiol groups during electrophoresis. The second dimension was performed in 12% acrylamide gels using an Ettan DALT II System (GEHealthcare, Madrid, Spain). The 2D-gels were stained with Flamingo™ Fluorescent Gel Stain (Bio Rad, Life Science, Barcelona, Spain) and scanned with a Typhoon FLA9500 (GE Healthcare, Madrid, Spain). For each independent experiment, 2-DE for protein extracts from SAT and VAT were processed in parallel to guarantee maximum comparability. Analyses investigating differences in protein patterns were performed with the PD-Quest 8.0 (BioRad, Life Science, Barcelona, Spain), using a single master that included all gels of each independent experiment. Each spot was assigned a relative value corresponding to the single spot volume compared to the volume of all spots in the gel, following background extraction and normalization between gels.

### 4.5. Western Blot Analysis

Protein was extracted using RIPA buffer (50 mM Tris–HCl pH 8, 150 mM NaCl, 1% NP-40, 0.5% sodium Deoxycholate, 0.1% SDS) or from 48 h cell supernatant. Protein concentrations were measured with Pierce BCA Protein Assay Kit (ThermoScientific, Barcelona, Spain). Twenty-five micrograms of protein were resolved by 1-DE under reducing conditions onto 10% SDS–PAGE gels and electrotransferred to nitrocellulose membranes. After blocking for non-specific binding, membranes were incubated with primary antibody; kazrin and beta-actin. Band detection was performed using a chemiluminescent substrate dye (SuperSignal^®^ West Dura Extended Duration Substrate, Thermo Scientific, Waltham, MA, USA) and a molecular imager ChemiDoc XRS System, Universal Hood II (BioRad, Hercules, CA, USA). Band quantification was performed with Quantity-One software (BioRad laboratories, Hercules, CA, USA). Protein load was normalized with beta actin.

### 4.6. Mass Spectrometry Analysis

As previously described [55], proteins were identified after in-gel tryptic digestion and extraction of peptides from the gel’s pieces, by matrix-assisted laser desorption/ionization time-of-flight (MALDI-TOF) using an AutoFlex III Smartbeam MALDI-TOF/TOF (Bruker Daltonics, Barcelona, Spain). Samples were applied to Prespotted AnchorChip plates (Bruker Daltonics, Barcelona, Spain) surrounding calibrates provided on the plates. Spectra were acquired with flexControl on reflector mode, (mass range 850–4000 m/z, reflector 1: 21.06 kV; reflector 2: 9.77 kV; ion source 1 voltage: 19 kV; ion source 2: 16.5 kV; detection gain 2.37×) with an average of 3500 added shots at a frequency of 200 Hz. Each sample was processed with flexAnalysis (version 3.0, Bruker Daltonics) considering a signal-to-noise ratio over 3, applying statistical calibration and eliminating background peaks. For identification, peaks between 850 and 1000 were not considered, as in general only matrix peaks are visible on this mass range. After processing, spectra were sent to the interface BioTools (version 3.2, Bruker Daltonics) and MASCOT search on Swiss-Prot 57.15 database was performed [Taxonomy: Homo Sapiens, Mass Tolerance 50 to 100, up to 2 miss cleavage, Global Modification: Carbamidomethyl (C), Variable Modification: Oxidation (M)]. Identification was accepted with a score higher than 56.

### 4.7. Quantification of Serum Protein Levels

A multiplexed bead-based immunoassay was utilized for the simultaneous measurement of different proteins. A 37-plex assay was performed to measure inflammation-related proteins (171AL001M, Bio-Rad, Life Science, Barcelona, Spain), a 10-plex assay for the measure of T2DM-related proteins (171A7001M, Bio-Rad, Life Science, Barcelona, Spain) and finally a 2-plex assay was used to measure adipsin and adiponectin due to the different dilutions required (171A7002M, Bio-Rad, Life Science, Barcelona, Spain). Multiplex assays are based on an internal color-coded bead that is coupled to analyze specific antibodies, allowing for the simultaneous measurement of multiple analyses in the same well. The assay was performed according to the supplier’s instructions (Bio Rad, Life Science, Barcelona, Spain).

### 4.8. In Silico Analysis

Differentially expressed proteins were employed to build protein–protein interaction (PPI) networks using the STRING database interaction data [56,57]. The maximum number of additional interactions was set to 10, and only interactions with a confidence score above 0.6 were considered. The resulting PPI networks were analyzed for Gene Ontology biological process terms enrichment. All PPI networks were built and analyzed using Cytoscape 3.0 software [58]. Thereafter, the influence of VAT and SAT proteomes’ composition on serum adipokine signature was evaluated following a correlation approach. To this end, a heatmap was built through the calculation of Spearman’s Rho correlation coefficient between every AT protein/adipokine pair. Hierarchical clustering was then applied to both rows and columns, to identify regulatory modules. Correlation analysis and heatmaps were conducted and analyzed with RStudio, using the R packages ‘stats’ and ‘ComplexHeatmap’ [59].

### 4.9. Statistical Analysis

Data were expressed as mean ± standard error of the mean (SEM), unless stated otherwise. Differences in protein levels between SAT and VAT, and between diabetic and non-diabetic individuals, were assessed using non-parametric tests (i.e., Wilcoxon or Mann–Whitney U tests). The level of significance was set at *p* < 0.05. All analyses were conducted with StatView 5.0 software.

## 5. Conclusions

In summary, in the present study, we have performed for the first time the parallel study of the AT (two depots, subcutaneous and visceral) and the serum protein profile of the same individuals with obesity and with or without T2DM, providing novel information of T2DM on visceral adiposity proteostasis. The impact of these metabolic conditions on the resident stem cells in these two depots may affect their multipotency capacity for regeneration. Additionally, we have also demonstrated for the first time the presence of KAZN in both adipose tissue depots, highlighting the need to further investigate adipose tissue composition, its paracrine regulation on the diverse body tissues and organs and the function of adipose cells.

## Figures and Tables

**Table 1:** Differential serum-circulating protein concentration due to obesity and T2DM.

	Effect of T2DM	Effect of Obesity	Effect of Obesity and T2DM
	OB-T2DM vs. OB-nonT2DM	OB-nonT2DM vs. nonOB-nonT2DM	OB-T2DM vs. nonOB-nonT2DM
leptin (ng/mL)	0.713	**<0.01**	**<0.01**
insulin (pg/mL)	0.52	**0.01**	**<0.01**
chitinase 3-like 1 (ng/mL)	**0.01**	0.66	**<0.01**
C-peptide (ng/mL)	0.86	**0.03**	**<0.01**
TNF-R1 (ng/mL)	**<0.01**	**0.03**	**<0.01**
ghrelin (pg/mL)	0.82	**<0.01**	**<0.01**
GLP-1 (pg/mL)	0.18	**0.01**	**<0.01**
glucagon (pg/mL)	0.08	**0.03**	**<0.01**
IL-6Ra (ng/mL)	**0.04**	**0.04**	0.84
TWEAK/TNFSF12 (pg/mL)	0.33	**0.03**	0.16
adiponectin (μg/mL)	0.75	**0.01**	**<0.01**
osteocalcin (ng/mL)	0.33	**<0.01**	**0.01**
MMP-2 (ng/mL)	0.59	**<0.01**	**<0.01**
PAI-1 (ng/mL)	0.96	**0.05**	0.16
GIP (pg/mL)	0.71	0.06	**0.02**
adipsin (μg/mL)	**<0.01**	0.07	**0.01**
TNF-R2 (pg/mL)	0.03	0.41	0.10
BAFF/TNFSF13B (ng/mL)	**0.05**	0.55	0.06
OPN (ng/mL)	**0.05**	0.16	0.58
MMP-3 (ng/mL)	**0.03**	0.24	0.26
IL-8 (pg/mL)	**0.05**	0.43	0.08

Statistical significance was accepted at *p* ≤ 0.05; red means that it is increased and green that it is decreased versus its direct comparator.

**Table 2:** Significance of changes in differential protein expression of SAT versus VAT in obese individuals (with and without T2DM); obese without T2DM individuals (OB-nonT2DM) and obese with T2DM individuals (OB-T2DM).

		VAT vs. SAT					
		ALL (*n* = 12)		OB-nonT2DM (*n* = 6)		OB-T2DM (*n* = 6)	
Protein Name	Gene	logFC	*p* Value	logFC	*p* Value	logFC	*p* Value
ATP-binding cassette sub-family G member 8	ABCG8	0.49	**0.01**	0.61	0.06	0.01	0.31
Annexin 1	ANXA1	0.61	**0.04**	0.23	0.69	1.07	**0.03**
Apolipoprotein A1	APOA1	−0.23	0.47	−0.16	0.44	0.41	**0.03**
Apo E	APOE	−0.36	0.38	−0.36	0.38	−2.25	**0.03**
N(G)-dimethylarginine dimethylaminohydrolase 2	DDHA2	0.64	**0.00**	0.78	**0.03**	0.13	0.13
Fumarylacetoacetase	FAH	−0.81	**0.05**	−1.02	0.06	−0.99	0.31
Fibrinogen gamma chain	FGG	1.63	**0.00**	1.79	**0.03**	1.15	**0.03**
Ferritin light chain	FTL	1.23	**0.05**	1.26	**0.03**	0.13	0.63
Glutathione S-transferase P	GSTP1	0.56	**0.02**	0.88	**0.03**	0.19	0.81
Hemopexin	HPX	−0.17	**0.03**	−0.06	0.31	−0.33	0.06
Kazrin	KAZN	2.19	**0.00**	1.21	**0.03**	3.74	0.06
Inorganic pyrophosphatase	PPA2	0.37	0.20	0.58	**0.03**	0.20	1.00
Transmembrane and coiled-coil domain-containing protein 7	TMCO7	0.56	**0.00**	1.03	0.13	0.40	**0.03**

*p* value < 0.05 is considered significant and marked in bold. Red proteins up-regulated in VAT. Green proteins down-regulated in VAT.

**Table 3:** Top 5 Gene Ontology biological process enrichments for visceral versus subcutaneous adipose tissue differential expression network. FDR, false discovery rate.

Top 5 Gene Ontology Biological Process Enrichments		
ID	Term	FDR
GO.0002576	Platelet degranulation	7.10 × 10^−8^
GO.0046903	Secretion	1.08 × 10^−6^
GO.0045055	Regulated exocytosis	4.87 × 10^−6^
GO.0032940	Secretion by cell	4.87 × 10^−6^
GO.0009611	Response to wounding	9.12 × 10^−6^

**Table 4:** Correlation between protein expression and different biochemical parameters. Direct regression in green and inverse regression in red.

Biochemical Parameters	Protein	SAT		VAT	
		R^2^	*p*	R^2^	*p*
**Glucose**	alcohol DH [NADP+]	0.039	0.638	0.392	**0.05**
	apolipoprotein E	0.139	0.53	0.635	**0.03**
	protein disulfide isomerase A3	0.389	**0.04**	0.101	0.34
**Tryglicerides**	annexin A5	0.017	0.7	0.385	**0.04**
	apolipoprotein A1	0.291	0.08	0.331	**0.03**
	breast carcinoma-amplified seq-1	0.558	**<0.01**	0.004	0.82
	haptoglobin	0.08	0.39	0.371	**0.04**
	hemopexin	0.477	**0.01**	0.216	0.15
	retinal DH	0.412	**0.03**	0.113	0.31
	protein disulfide isomerase A3	0.203	0.61	0.476	**0.02**
	paroxiredoxin 2	0.496	**0.02**	0.03	0.61
	ribonuclease inhibitor	0.552	**<0.01**	0.153	0.23
**HDL**	alpha-1 antitrypsin	0.414	**0.04**	0.116	0.33
	annexin A5	0.001	0.82	0.459	**0.03**
	D2 dopamine receptor	0.495	**0.02**	0.066	0.47
	L-lactate DH A chain	0.454	**0.03**	0.551	**0.01**
**LDL**	actin. Aortic smooth muscle	0.652	**<0.01**	0.385	**0.04**
	annexin A3	0.41	**0.04**	0.009	0.77
	apolipoprotein E	0.551	0.15	0.636	**0.03**
	breast carcinoma-amplified seq-1	0.544	**0.01**	0.02	0.69
	creatine kinase B-type	0.844	**<0.01**	0.001	0.91
	glycerol-3-phosphate DH [NAD+]	0.398	**0.03**	0.001	0.92
	haptoglobin	0.003	0.87	0.373	**0.04**
	HSP 60	0.396	**0.03**	0.042	0.54
	intelectin-1	0.399	**0.03**	0.234	0.132
	retinal DH	0.67	**<0.01**	0.029	0.62
	paroxiredoxin 2	0.588	**0.01**	0.082	0.39
	serine/threonine protein kinase	0.753	**<0.01**	0.044	0.53
	tropomyosin beta chain	0.137	0.29	0.424	**0.03**
**Cholesterol**	14-3-3 Protein alpha/beta	0.479	**0.01**	0.04	0.55
	14-3-3 Protein gamma	0.57	**<0.01**	0.247	0.24
	14-3-3 Protein zeta/delta	0.514	**0.01**	0.338	0.06
	26S protein regulatory subunit 6B	0.179	0.29	0.696	**<0.01**
	ADP/ATP Translocase 3	0.38	**0.04**	0.092	0.39
	alpha-1 antitrypsin	0.582	**<0.01**	0.003	0.87
	annexin A5	0.001	0.8	0.359	**0.05**
	apolipoprotein A1	0.022	0.66	0.44	**0.02**
	collagen alpha-1 (XIII) chain	0.39	**0.05**	0.496	**0.02**
	fibrinogen gamma chain	0.117	0.3	0.378	**0.04**
	haptoglobin	0.535	**0.01**	0.213	0.15
	hemopexin	0.196	0.17	0.446	**0.02**
	HSP 60	0.002	0.88	0.463	**0.03**
	serine/threonine protein kinase	0.251	0.11	0.433	**0.02**

**Table 5:** Significance of changes in differential protein expression in T2DM versus non-T2DM individuals in VAT and SAT adipose.

		T2DM vs. nonT2DM			
		SAT		VAT	
Protein Name	Gene	logFC	*p* Value	logFC	*p* Value
Alpha-1B-glycoprotein	A1BG	0.19	0.54	1.27	**0.01**
Actin. aortic smooth muscle	ACTA2	−2.62	**0.03**	−1.60	0.39
Albumin	ALB	3.26	**0.02**	2.69	0.06
Annexin A3	ANXA3	−0.99	**0.00**	−1.23	**0.00**
Annexin A8-like protein 2	ANXA8L1	−0.69	0.80	−1.48	**0.00**
Apolipoprotein A1	APOA1	−1.64	**0.02**	−1.06	0.70
Actin-related protein 2	ARP2	−0.98	0.48	−0.43	**0.02**
Collagen alpha-1(XIII) chain	COLBA1	−2.16	**0.13**	−2.85	**0.05**
Cytochrome c oxidase subunit 7A-related protein, mitochondrial	COX7A2L	1.40	**0.04**	0.54	0.25
Fibrinogen gamma chain	FGG	−0.74	0.13	−1.38	**0.01**
Haptoglobin	HP	1.25	**0.04**	1.60	**0.04**
Hemopexin	HPX	0.69	0.24	0.42	**0.04**
HSP 27	HSPB1	−3.18	0.06	−1.72	**0.00**
HSP 60	HSPD1	−0.88	**0.00**	−0.39	0.82
Protein disulfide isomerase	PD1	1.65	0.41	1.41	**0.00**
Selenium-binding protein 1	SELENBP1	0.95	**0.00**	0.54	0.70
Antithrombin III	SERPINC1	1.13	0.48	3.14	**0.01**
Solute carrier family 25 member 6	SLC25A6	1.18	0.06	1.07	**0.00**
26S protease regulatory subunit 6B	PSMC4	−1.17	**0.05**	−1.18	0.26
Transthyretin	TTR	0.31	0.25	0.44	**0.03**
Ubiquitin carboxyl-terminal hydrolase isozyme 1	UCHL1	1.22	**0.04**	0.36	0.39
Vimentin	VIM	−0.84	0.39	−0.14	0.82
14-3-3 protein b/a	YWHAB	−0.98	**0.03**	−1.40	**0.00**
14-3-3 protein gamma	YWHAG	−0.20	0.48	−0.68	0.06
14-3-3 protein zeta/delta	YWHAZ	−1.63	**0.03**	−1.37	**0.04**

*p* value < 0.05 is considered significant. Red proteins up-regulated and green proteins down-regulated in T2DM versus nonT2DM.

**Table 6:** Top 5 Gene Ontology biological process enrichments for type 2 diabetes mellitus differential expression networks for visceral and subcutaneous white adipose tissue depots.

Top 5 Gene Ontology Biological Process Enrichments					
VAT T2DM vs. nonT2DM			SAT T2DM vs. nonT2DM		
ID	Term	FDR	ID	Term	FDR
GO.0006810	Transport	4.62 × 10^−10^	GO.0006950	Response to stress	8.64 × 10^−8^
GO.0045055	Regulated exocytosis	8.62 × 10^−8^	GO.0051234	Establishment of localization	2.20 × 10^−7^
GO.0002576	Platelet degranulation	8.62 × 10^−8^	GO.0009894	Regulation of catabolic process	3.29 × 10^−7^
GO.0046903	Secretion	2.88 × 10^−7^	GO.0006810	Transport	1.19 × 10^−6^
GO.0016192	Vesicle-mediated transport	2.88 × 10^−7^	GO.0031329	Regulation of cellular catabolic process	1.25 × 10^−6^

**Table 7:** Demographic description and clinical parameters of the individuals included in the proteomic analysis.

	nonT2DM (N = 6)	T2DM (N = 6)
Age	50 ± 3	53 ± 3
BMI (kg/m^2^)	43.6 ± 1.0	43.3 ± 1.3
Gender (M/W)	3/3	3/3
CV Risk factors	1	3–5
FBG (mg/dL)	127.6 ± 19.5	149.4 ± 13.6
TG (mg/dL)	173.2 ± 37.8	170.8 ± 12.7
Cholesterol (mg/dL)	168.4 ± 12.2	197.8 ± 21.9
HDL (mg/dL)	33.5 ± 4.0	39.5 ± 8.4
LDL (mg/dL)	226.8 ± 57.1	170.2 ± 40.8
Urea (mg/dL)	28.2 ± 5.1	47.3 ± 18.6
Total proteins (g/dL)	6.9 ± 0.3	7.5 ± 0.4
AST UL	25.0 ± 4.6	17.6 ± 2.8
ALT UL	20.8 ± 4.6	15.4 ± 2.8
AST/ALT	0.9 ± 0.2	1.0 ± 0.1
Creatinine	1.0 ± 0.1	1.1 ± 0.2

Data expressed as mean value ± SEM. Abbreviations: ALT: alanine transaminase; AST: aspartate aminotransferase; BMI: body-mass index; M: men; W: women; CV: cardiovascular; FBG: fasting blood glucose; HDL: high-density lipoprotein; LDL: low-density lipoprotein; T2DM: type 2 diabetes mellitus; TG: triglycerides.

## Data Availability

The data presented in this study are available on request from de corresponding authors.

## Supplementary Materials

The following supporting information can be downloaded at: https://www.mdpi.com/article/10.3390/ijms24010827/s1.

## Abbreviations

AT: adipose tissue; ASCs: adipose stem cells; nonOB: non-obese; OB: obese; nonT2DM: non-type 2 diabetes mellitus; SAT: subcutaneous adipose tissue; T2DM: type 2 diabetes mellitus; VAT: visceral adipose tissue.
