# Supplementary material for: Type 2 Diabetes in Obesity: A Systems Biology Study on Serum and Adipose Tissue Proteomic Profiles
Source: Int J Mol Sci. 2023 Jan 3;24(1):827. doi: 10.3390/ijms24010827 (PMC9821208; doi:10.3390/ijms24010827)
Supplement: Supplementary file 1 [file ijms-24-00827-s001.zip › ijms-2082656-supplementary.pdf]

Supplemental data

Type 2 Diabetes in Obesity: A Systems Biology Study on Serum and Adipose Tissue Proteomic Profiles

Gemma Arderiu [1,2,*,†], Guiomar Mendieta [3,*], Alex Gallinat [1], Carmen Lambert [1,4], Alberto Díez-Caballero [5], Carlos Ballesta [5] and Lina Badimon[1,2,†]

* Equal contribution

1 Cardiovascular-Program, Institut d'Investigació Biomèdica Sant Pau (IIB Sant Pau), Barcelona, Spain
2 Centro de Investigación Biomédica en Red de Enfermedades Cardiovasculares (CiberCV), Barcelona, Spain
3 Centro Nacional de Investigaciones Cardiovasculares (CNIC), Madrid, Spain
4 Actual position in IPSA-Instituto de investigación Sanitaria del Principado de Asturias, Oviedo, Spain
5 Centro Médico Teknon, Barcelona, Spain

Supplementary Table S1. Differential serum-circulating protein concentration (pg/mL) due to obesity with and without diabetes. Data represent mean ± SEM. (n.d not detectable; P<0.05). In green- lower level and in red- higher level.

	nOB-nT2DM	OB-nT2DM	OB-T2DM	OB-nT2DM *vs* nOB-nT2DM	OB-T2DM *vs* nOB-nT2DM	OB-T2DM *vs* OB/non-T2DM
**Leptin (ng/ml)**	4.1 ± 1.3	23.7 ± 4.8	21.3 ± 4.5	**<0.01**	**<0.01**	0.713
**Insulin (pg/ml)**	395.6 ± 70.8	1004.9 ± 308.8	1100.3 ± 241.1	**0.01**	**<0.01**	0.52
**Chitinase 3-like 1 (ng/ml)**	15.9 ± 1.2	20.0 ± 3.0	34.3 ± 4.7	0.66	**<0.01**	**0.01**
**C-peptide (ng/ml)**	0.8 ± 0.1	1.6 ± 0.2	1.6 ± 0.2	**0.03**	**<0.01**	0.86
**TNF-R1 (ng/ml)**	1.2 ± 0.1	1.5 ± 0.1	2.5 ± 0.2	**0.03**	**<0.01**	**<0.01**
**Ghrelin (pg/ml)**	95.2 ± 2.1	164.0 ± 44.9	126.7 ± 13.0	**<0.01**	**<0.01**	0.82
**GLP-1 (pg/ml)**	195.2 ± 8.7	228.7 ± 7.8	248.1 ± 9.9	**0.01**	**<0.01**	0.18
**Glucagon (pg/ml)**	1064.9 ± 15.7	1123.8 ± 18.5	1150.9 ± 13.4	**0.03**	**<0.01**	0.08
**IL-6Ra (ng/ml)**	16.4 ± 1.0	13.4 ± 2.3	18.4 ± 3.2	**0.04**	0.84	**0.04**
**TWEAK/TNFSF12 (pg/ml)**	564.8 ± 38.4	420.0 ± 43.8	489.9 ± 57.2	**0.03**	0.16	0.33
**Adiponectin (µg/ml)**	75.1 ± 5.6	54.2 ± 4.6	50.8 ± 3.0	**0.01**	**<0.01**	0.75
**Osteocalcin (ng/ml)**	7.6 ± 0.7	3.6 ± 0.4	4.9 ± 1.0	**<0.01**	**0.01**	0.33
**MMP-2 (ng/ml)**	40.2 ± 2.5	21.5 ± 3.8	22.6 ± 2.8	**<0.01**	**<0.01**	0.59
**PAI-1 (ng/ml)**	63.6 ± 6.1	45.2 ± 7.3	44.7 ± 10.6	**0.05**	0.16	0.96
**GIP (pg/ml)**	181.6 ± 14.5	404.9 ± 167.8	230.4 ± 16.2	0.06	**0.02**	0.71
**Adipsin (µg/ml)**	1.2 ± 0.1	1.3 ± 0.1	1.8 ± 0.1	0.07	**0.01**	**<0.01**
**TNF-R2 (pg/ml)**	760.2 ± 72.8	699.4 ± 65.9	1081.7 ± 128.0	0.41	0.10	**0.03**
**BAFF/TNFSF13B (ng/ml)**	9.0 ± 1.1	8.3 ± 0.7	9.9 ± 0.5	0.55	0.06	**0.05**
**Osteopontin (OPN) (ng/ml)**	23.2 ± 4.6	15.2 ± 2.3	22.6 ± 2.2	0.16	0.58	**0.05**
**MMP-3 (ng/ml)**	6.1 ± 0.7	5.0 ± 0.7	9.7 ± 2.7	0.24	0.26	**0.03**
**IL-8 (pg/ml)**	117.3 ± 19.9	126.5 ± 10.1	82.2 ± 9.1	0.43	0.08	**0.05**
**APRIL/TNFSF13 (ng/ml)**	41.5 ± 9.9	66.0 ± 9.2	78.2 ± 15.3	0.10	0.07	0.73
**CD163 (ng/ml)**	110.0 ± 12.2	142.6 ± 16.3	182.6 ± 30.5	0.17	0.09	0.50
**IL-35 (pg/ml)**	271.5 ± 36.2	393.8 ± 43.0	323.1 ± 73.0	0.07	0.77	0.30
**IL-12(p40) (pg/ml)**	205.0 ± 20.4	179.6 ± 25.1	188.1 ± 24.8	0.12	0.43	0.92

**IL-32 (pg/ml)**	264.9 ± 52.2	209.9 ± 39.0	257.5 ± 44.7	0.21	0.77	
**MMP-1 (ng/ml)**	2.5 ± 0.6	3.5 ± 0.9	1.8 ± 0.3	0.23	0.58	0.10
**LIGHT/TNFSF14 (pg/ml)**	57.5 ± 17.3	40.9 ± 18.0	78.7 ± 40.8	0.29	>0.99	0.72
**Pentraxin-3 (ng/ml)**	12.8 ± 1.6	11.8 ± 1.9	12.8 ± 1.8	0.32	0.71	0.66
**IL-10 (pg/ml)**	23.3 ± 2.1	30.4 ± 5.7	28.8 ± 2.8	0.34	0.17	0.97
**IL-26 (pg/ml)**	1157.2 ± 258.9	771.2 ± 85.0	922.2 ± 213.7	0.36	0.46	>0.99
**Visfatin (pg/ml)**	6.5 ± 0.4	7.8 ± 0.9	8.1 ± 0.7	0.38	0.12	0.44
**gp130 (ng/ml)**	94.2 ± 8.6	91.8 ± 11.3	117.5 ± 18.8	0.44	0.79	0.66
**IL-2 (pg/ml)**	45.2 ± 6.2	60.1 ± 14.3	54.6 ± 11.3	0.45	0.72	0.83
**CD30/TNFRSF8 (pg/ml)**	794.9 ± 80.5	743.6 ± 85.2	852.4 ± 94.7	0.48	0.66	0.36
**IL-20 (pg/ml)**	38.8 ± 5.9	169.2 ± 131.6	39.1 ± 5.0	0.59	0.88	0.54
**IL-27(p28) (pg/ml)**	81.4 ± 18.7	86.8 ± 24.4	92.4 ± 25.3	0.68	0.82	0.49
**TSLP (pg/ml)**	23.9 ± 1,9	24.1 ± 2.3	29.1 ± 2.9	0.78	0.19	0.07
**IL-34 (pg/ml)**	678.4 ± 214.6	641.9 ± 183.1	757.2 ± 139.0	0.80	-	0.66
**IFN-g (pg/ml)**	58.4 ± 6.8	60.8 ± 7.6	56.0 ± 6.2	0.91	0.93	0.84
**IL-11 (pg/ml)**	26.2 ± 20.4	6.3 ± 0.6	6.7 ± 0.5	0.94	0.37	0.48
**Resistin (pg/ml)**	11.1 ± 1.3	11.0 ± 1.0	12.1 ± 1.5	0.95	0.92	0.51
**IL-22 (pg/ml)**	131.2 ± 35.1	153.9 ± 11.5	382.8 ± 0.0	>0.99	0.16	0.32
**IL-12(p70) (pg/ml)**	9.1 ± 0.0	n.d.	10.3 ± 0.0	-	0.32	-
**IL-29/IFN-l1**	n.d.	65.5 ± 0.0	141.3 ± 48.8	-	>0.99	-
**IL-19**	n.d.	64.1 ± 0.0	83.5 ± 0.0	-	-	-
**IL-28A/IFN-12**	n.d.	n.d	n.d			
**IFN-a2**	n.d.	n.d	n.d			
**IFN-b**	n.d.	n.d	n.d			

Supplementary Table S2. Protein expression of SAT and VAT in obese class III patients with or without T2DM. Data represent mean ± SEM.
P value <0.05 is considered significant and marked in bold.

Gene	Protein	OB-nT2DM		OB-T2DM		OB	OB/non - T2DM	OB/ T2DM
		SAT	VAT	SAT	VAT	SAT *vs* VAT		
YWHAB	14-3-3 protein b/a	59.5 ± 7.8	49.0 ± 5.6	30.7 ± 5.6	18.0 ± 4.0	**0.05**	0.116	0.173
YWHAG	14-3-3 protein gamma	107.0 ± 17.1	142.0 ± 20.5	89.6 ± 16.8	93.6 ± 12.9	0.239	0.075	0.6
YWHAZ	14-3-3 protein zeta/delta	74.6 ± 11.8	250.9 ± 27.7	35.4 ± 12.1	140.8 ± 20.1	0.583	0.249	0.6
YWH	14-3-3 protein Total	241.1 ± 34.1	59.9 ± 8.6	155.8 ± 33.4	29.1 ± 7.4			
PSMC4	26S protease regulatory subunit 6B	21.5 ± 1.8	19.8 ± 2.8	11.4 ± 3.3	14.5 ± 7.1	0.953	0.752	0.593
GRP78	78kDa glucose-related protein	43.1 ± 5.2	56.6 ± 13.4	56.6 ± 10.8	64.7 ± 11.6	0.182	0.116	0.753
ACTA2	Actin. aortic smooth muscle	457.6 ± 83.0	488.5 ± 266.3	170.1 ± 85.2	90.7 ± 26.6	0.388	0.917	0.345
ACTB	Actin. cytoplasmic 1	723.0 ± 194.5	884.0 ± 473.9	1292.1 ± 265.6	979.8 ± 113.3	0.433	0.917	0.173
ARP2	Actin-related protein 2	21.3 ± 8.1	18.2 ± 3.5	10.2 ± 2.5	8.9 ± 1.6	0.213	0.753	0.043
SCL25A6	ADP/ATP Translocase 3	22.9 ± 0.9	32.6 ± 3.5	59.2 ± 14.1	69.8 ± 8.4	0.182	0.116	0.686
ABCG8	ATP-binding cassette sub-family G member 8	2.7 ± 0.4	10.8 ± 3.9	5.3 ± 1.1	14.4 ± 2.5	0.016	0.043	0.249
ADH6	Alcohol DH [NADP+]	10.4 ± 1.6	65.6 ± 17.8	12.3 ± 1.7	84.3 ± 20.3	0.674	0.285	0.225
ALDH	Aldehyde DH	51.3 ± 18.0	1030.4 ± 150.3	54.0 ± 16.6	1004.5 ± 113.4	0.213	0.225	0.345
SERPIN	Alpha-1-antitrypsin	706.5 ± 92.8	14.6 ± 2.1	894.9 ± 152.8	33.3 ± 3.3	0.117	0.075	0.917
A1BG	Alpha-1B-glycoprotein	14.8 ± 3.5	44.1 ± 14.0	22.6 ± 6.2	90.8 ± 31.1	0.508	0.686	0.225
LRPAP1	Alpha-2-macroglobulin receptor-associated protein	36.4 ± 13.9	42.0 ± 7.0	57.9 ± 9.4	13.7 ± 2.9	0.333	0.686	0.08
ANXA8L1	Annexin A8-like protein 2	19.5 ± 7.7	30.9 ± 9.5	11.9 ± 3.3	32.9 ± 3.2	0.345	0.18	0.593
ANXA1	Annexin A1	29.0 ± 6.6	20.6 ± 2.5	16.1 ± 3.5	9.5 ± 1.2	0.041	0.6	**0.028**
ANXA3	Annexin A3	20.7 ± 2.8	289.9 ± 32.3	9.7 ± 1.0	240.1 ± 35.1	0.929	0.893	0.917
ANXA5	Annexin A5	364.6 ± 74.6	178.2 ± 46.4	323.8 ± 64.6	132.7 ± 25.1	0.695	0.345	0.463
ANXA6	Annexin A6	124.2 ± 31.7	10.6 ± 3.5	156.8 ± 38.4	131.9 ± 19.3	0.182	0.6	0.917
SERPINC1	Antithrombin III	43.4 ± 29.3	7.2 ± 1.5	86.2 ± 14.7	6.0 ± 1.8	0.423	0.686	0.116
APOE	Apo E	10.5 ± 2.3	726.9 ± 196.3	21.2 ± 3.1	503.2 ± 137.7	0.273	-	0.273
APOA1	Apolipoprotein A1	1124.0 ± 317.4	88.2 ± 14.2	324.7 ± 70.0	66.6 ± 12.1	0.433	0.345	**0.028**
ATP5F1B	ATP synthase subunit beta. mitochondrial	78.6 ± 11.3	4.1 ± 0.8	53.7 ± 10.3	6.6 ± 1.6	0.347	0.463	0.753
BLVRA	Biliverdin reductase A	32.7 ± 17.2	10.0 ± 2.9	27.4 ± 5.4	3.8 ± 0.9	0.465	0.465	
BCAS1	Breast carcinoma-amplified sequence 1	139.9 ± 82.3	45.5 ± 11.5	93.4 ± 32.6	99.9 ± 47.2	0.182	0.173	0.5
COLBA1	Collagen alpha-1(XIII) chain	32.9 ± 11.5	27.1 ± 7.1	11.8 ± 7.4	10.7 ± 7.6	0.477	0.753	**0.08**
CKB	Creatine kinase B-type	45.5 ± 23.4	45.7 ± 9.8	26.7 ± 5.6	46.6 ± 7.2	0.248	0.917	**0.043**
COX7A2L	Cytochrome c oxidase subunit 7A-related protein. Mitochondrial	155.9 ± 60.0	204.5 ± 33.4	381.7 ± 56.6	282.7 ± 40.8	0.441	0.068	0.08
DRD2	D2 Dopamine receptor	8.1 ± 1.1	9.7 ± 1.3	11.6 ± 2.1	9.9 ± 1.9	0.875	0.463	0.116
ECH1	Delta(3.5)-Delta(2.4)-dienoyl-CoA isomerase. Mitochondrial	17.1 ± 3.8	16.2 ± 4.7	19.6 ± 8.8	28.5 ± 11.5	0.499	0.715	0.109
ALAD	Delta-aminolevulinic acid dehydratase	107.2 ± 78.0	26.6 ± 7.1	28.5 ± 5.3	25.9 ± 4.0	0.155	0.225	0.6
ENOA	ENO A	54.8 ± 14.8	50.7 ± 6.9	34.8 ± 4.2	39.7 ± 3.6	0.754	0.753	0.463
FTL	Ferritin ligth chain	56.2 ± 30.4	101.0 ± 40.7	118.5 ± 42.6	134.1 ± 25.5	0.051	**0.028**	0.5
FGG	Fibrinogen gamma chain	65.1 ± 19.6	208.0 ± 45.0	28.2 ± 6.9	74.3 ± 14.5	**0.002**	**0.028**	**0.028**
GSTP1	Glutathione S-transferase P	83.7 ± 10.4	165.2 ± 23.4	144.9 ± 24.4	171.9 ± 20.0	**0.021**	0.028	0.686
GPX3	Glutathione peroxidase	27.7 ± 6.4	25.6 ± 3.4	23.3 ± 3.0	23.5 ± 2.6	0.79	0.917	0.686
GPSA	Gycerlo-3-phosphate dehydrogenase [NAD+]	401.0 ± 74.5	368.5 ± 72.0	366.1 ± 71.1	239.8 ± 33.7	0.308	0.917	0.173
HP	Haptoglobin	159.0 ± 31.5	203.9 ± 76.4	336.8 ± 71.5	405.0 ± 52.0	0.53	0.917	0.463
HSPA1B	Heat shock 70kDa protein 1A/1B	79.9 ± 8.4	85.7 ± 21.8	73.1 ± 31.6	100.1 ± 20.5	0.374	0.345	0.345
HSC70	Heat shock cognate 71kDa protein	81.4 ± 16.7	85.4 ± 16.4	133.8 ± 23.1	92.6 ± 18.3	0.433	0.463	0.249
HPX	Hemopexin	408.4 ± 141.3	266.3 ± 40.3	508.6 ± 90.2	400.9 ± 56.8	**0.028**	0.249	**0.046**
HSPB1	HSP 27	90.2 ± 32.4	86.9 ± 12.5	21.8 ± 10.7	27.2 ± 6.	0.308	0.463	0.463
HSPD1	HSP 60	51.5 ± 19.8	43.3 ± 5.9	17.7 ± 0.9	71.5 ± 36.3	0.084	0.600	**0.046**
IRF2BP1	I2BP1	210.3 ± 50.6	196.7 ± 40.1	235.8 ± 62.2	305.4 ± 50.4	0.583	0.753	0.463
PPA2	Inorganic pyrophosphatase	29.6 ± 3.0	46.1 ± 7.1	43.7 ± 9.4	40.4 ± 4.6	0.182	**0.028**	0.917
ITLN1	Intelectin-1	17.2 ± 6.8	22.8 ± 6.4	17.1 ± 3.8	26.1 ± 5.3	0.239	0.6	0.249
KAZN	Kazrin	30.3 ± 9.2	94.3 ± 29.2	16.4 ± 7.6	94.3 ± 28.9	**0.006**	**0.028**	**0.046**
LDHA	L-lactate dehydrogenase A chain	76.6 ± 17.5	56.6 ± 16.3	150.8 ± 44.5	140.1 ± 32.0	0.433	0.345	0.753
LDHB	L-lactate dehydrogenase B chain	100.8 ± 26.5	132.4 ± 67.8	105.3 ± 19.5	126.1 ± 15.9	0.938	0.6	0.463
DDHA2	N(G)-dimethylarginine dimethylaminohydrolase 2	17.5 ± 3.3	31.7 ± 4.9	27.9 ± 6.0	37.1 ± 6.8	**0.004**	0.028	0.08
PRDX1	Peroxiredoxin 1	18.7 ± 3.2	28.0 ± 2.2	14.2 ± 2.6	3.5 ± 0.8	>0.999	0.18	0.18

PRDX2	Peroxiredoxin 2	865.9 ± 354.0	515.8 ± 77.1	296.3 ± 138.0	269.8 ± 116.0	0.79	0.463	0.686
PRDX6	Peroxiredoxin 6	179.7 ± 27.8	265.5 ± 46.3	120.7 ± 40.2	172.2 ± 11.9	0.071	0.345	0.173
PD1	Protein disulfide isomerase	14.8 ± 1.2	18.2 ± 4.0	34.8 ± 9.6	45.4 ± 5.4	>0.999	0.999	0.999
PDIA3	Protein disulfide-isomerase A3	121.8 ± 19.0	189.8 ± 39.5	102.0 ± 19.4	118.4 ± 21.6	0.085	0.075	0.6
PK	Pyridoxal kinase	24.8 ± 5.5	24.2 ± 6.1	31.1 ± 8.6	34.7 ± 6.7	0.754	0.917	0.6
ALDH1A1	Retinal dehydrogenase 1	81.4 ± 32.4	88.1 ± 22.0	40.7 ± 14.9	70.6 ± 23.8	0.209	0.6	0.249
ARHGDIA	Rho GDP-dissotiation inhibitor	54.1 ± 13.1	51.3 ± 5.3	35.8 ± 12.7	39.7 ± 9.7	0.272	0.753	0.249
RNH1	Ribonuclease inhibitor	30.0 ± 6.3	46.3 ± 10.4	25.4 ± 3.4	25.4 ± 2.7	0.209	0.173	0.753
SBP1	Selenium-binding protein 1	57.3 ± 17.4	135.3 ± 28.1	116.2 ± 17.4	149.5 ± 20.2	**0.006**	**0.028**	0.116
ACPS	Serum amyloid P	36.9 ± 5.7	55.2 ± 6.7	35.6 ± 4.9	52.0 ± 4.3	**0.02**	0.046	0.500
OSK3	Serin/Threonine-protein kinase	96.9 ± 33.3	167.6 ± 38.3	90.9 ± 43.0	130.9 ± 25.5	0.155	0.225	0.345
PPP2CA	Serine/threonine-protein phosphatase 2A catalytic subunit alpha	32.2 ± 8.7	16.6 ± 1.2	13.6 ± 4.0	11.9 ± 2.1	0.347	0.249	0.917
TF	Serotransferrin	736.9 ± 181.6	1008.5 ± 217.4	1086.4 ± 298.3	922.5 ± 187.3	0.53	0.345	0.917
ACADS	Short-chain specific acyl-CoA dehydrogenase. mithocondrial	11.4 ± 2.7	9.1 ± 1.7	14.9 ± 3.8	22.2 ± 6.2	0.929	0.463	0.5
TTR	transhyretin	154.6 ± 21.4	165.1 ± 15.6	201.5 ± 23.0	215.6 ± 10.9	0.424	0.6	0.5
TMCO7	Transmembrane and coiled-coil domain-containing protein 7	3068.3 ± 735.3	3756.6 ± 1069.2	1891.4 ± 348.7	2927.8 ± 536.2	**0.005**	0.068	**0.028**
TPM2	Tropomyosin beta chain	52.8 ± 14.0	103.6 ± 33.7	64.3 ± 25.9	89.9 ± 12.9	0.11	0.249	0.225
UCHL1	Ubiquitin carboxyl-terminal hydrolase isozyme 1	28.6 ± 6.5	42.4 ± 10.9	56.8 ± 9.1	54.7 ± 9.9	0.239	0.116	0.753
VIM	Vimentin	257.8 ± 79.0	192.3 ± 59.4	147.6 ± 45.3	144.3 ± 15.3	0.695	0.173	0.463
GC	Vitamin D-binding protein	30.7 ± 16.9	102.5 ± 48.5	41.5 ± 2.0	71.6 ± 18.1	0.345	0.273	0.655
